# ACKNOWLEDGEMENTS

**DOI:** 10.2174/138920212803759686

**Published:** 2012-12

Bentham Science Publishers would like to thank and appreciate the co-operation from all reviewers for their constructive comments and feedback on the manuscripts submitted to **Current Genomics**. Their efforts have contributed greatly to the high quality and continuous growth of the journal. Given below is the list of reviewers who reviewed articles for the Journal during 2012:

1. **A. Carvalho**
2. **Al. Gjumrakch**
3. **B. Taylor**
4. **C. Pagnini**
5. **E. Corner**
6. **E. Reszka**
7. **J. Park**
8. **J. Badano**
9. **J. Duran**
10. **J. Zhua**
11. **Jun Qin**
12. **A. Liston**
13. **M. Szaumkessel**
14. **M. Haque**
15. **M. Harper**
16. **M. Kauffman.**
17. **M. Xiong**
18. **P. Adler**
19. **P. Herr**
20. **R. Chahwan**
21. **R. Kohli**
22. **S. Johnsen**
23. **U. Stelzl**

